# Evaluation of a multidisciplinary global health online course in Mexico

**DOI:** 10.1186/s41256-020-00179-8

**Published:** 2020-11-17

**Authors:** Héctor Carrasco, Patricia Fuentes, Itzel Eguiluz, Cesar Lucio-Ramírez, Sandra Cárdenas, Ilse Mariana Leyva Barrera, Manuel Pérez-Jiménez

**Affiliations:** 1 Tecnológico de Monterrey, Escuela de Medicina y Ciencias de la Salud, Monterrey, Mexico; 2 History of Science and Global Health and Health Policy, Harvard University, Cambridge, USA; 3 Vice-Rectory of Academic and Educational Innovation, Tecnológico de Monterrey, Monterrey Campus, Monterrey, Mexico

**Keywords:** Global Health Education, Global Health, LMICs, Global Health training

## Abstract

**Background:**

Global Health Education (GHE) focuses on training proactive global citizens to tackle health challenges in an increasingly interconnected and interdependent world. Studies show that health professionals in training have reported that GHE has improved their teamwork, responsiveness to contextual factors that impact health, and understanding of health systems; however, there is little research on the impact of GHE courses in undergraduate settings, especially in low and middle-income countries (LMICs).

**Methods:**

Our study analyzes a multidisciplinary online global health course at Tecnologico de Monterrey, México. We conducted a cross-sectional study with pre- and post-design. Students who took the multidisciplinary course of Global Health for Leaders in the Fall of 2019 (*n* = 153) and Spring of 2020 (*n* = 348) were selected for this study. Using a five-point Likert scale (strongly agree to strongly disagree), the survey assessed seven competencies as well as questions about course expectations, takeaways, and recommendations to improve the course. We performed descriptive statistical analyses comparing the combined pre-tests (from Fall and Spring cohorts) to the combined post-tests. Fisher’s exact test was used to compare the samples.

**Results:**

Of the 501 pre-course surveys administered, 456 responses were completed in the pre-course and 435 in the post-course (91% overall response rate). Only 8.7% of the respondents in the pre-course survey strongly agreed that they could describe fundamental aspects of global health such as the Millennium Development Goals or Sustainable Development Goals, in contrast to a 56% of the students who strongly agreed in the post-course survey (*p* < 0.001). Similar differences were captured in understanding the global burden of disease, social determinants of health, the effects of globalization in health, health systems’ goals and functions, and human rights. 38% felt that the course helped them develop a more empathetic perception of the suffering of others experiencing global health-related issues.

**Conclusion:**

In this study, we have presented our experience in teaching an online global health course for multidisciplinary undergraduates in a LMIC. The competencies reported by our students indicate that the course prepared them to confront complex global health issues.

**Supplementary Information:**

The online version contains supplementary material available at 10.1186/s41256-020-00179-8.

## Background

Global Health is concerned with health challenges with global impact, and it seeks global solutions through the power of academic research and science to promote health for all, and to improve health equity and reduce health disparities [1]. Global health challenges such as climate change, non-communicable chronic diseases (NCDs), antibiotic resistance, rapid urbanization, and infectious outbreaks such as COVID-19, disproportionately affect the poor, particularly in low- and middle-income countries (LMICs) [2, 3]. For example, Mexico has 61 million people living in poverty or extreme poverty, and high rates of obesity, diabetes, and adolescent pregnancy [4, 5]. Often, these health challenges are the result of large-scale global forces such as globalization -- understood as the unrestricted exchange of goods, services, infectious agents, and habits, which amplifies the market for harmful substances, enables the rapid proliferation of infectious diseases, and promotes unhealthy lifestyles [6] -- and neoliberalism, which advances free-market policies that result in privatization, deregulation, and limited government spending [7]. The impact of globalization and neoliberalism in health have been described elsewhere [7–10] and given their complexity, require comprehensive, multidisciplinary, and trans-sectoral responses. Thus, the field of global health emerged in response to growing health challenges and social inequalities that result from complex global forces [10].

Global Health Education (GHE) is part of the Global Citizenship Education movement proposed by the United Nations Educational, Scientific, and Cultural Organization (UNESCO) to train proactive global citizens in an increasingly interconnected and interdependent world [11]. GHE is uniquely suited to tackle current and future health issues because it is practice-oriented and competency-based [12–15]. The former challenges students to create and explore solutions to pressing global health problems by analyzing the connection between local factors and health challenges in a community, and ultimately bridging these observations with professionals who are already addressing health challenges [16]. Competency-based education trains professionals accountable to societal needs by developing observable and measurable optimal performance characteristics [17]. In contrast to traditional training approaches, health professionals in training have reported that GHE has improved their teamwork, responsiveness to contextual factors that impact health, and understanding of health systems [18, 19]. However, there is little research on the impact of GHE programs in undergraduate settings. Some authors claim that the potential impact includes a better understanding of cultural differences, more empathy towards underserved populations, and an increased “sense of idealism” to tackle health disparities [20]. Still, there is no evidence of these benefits reported from students in LMICs.

Despite the benefits of GHE and pervasive disparities in health and socioeconomic status among LMICs, GHE has made little headway. Currently, the most active and notable alliance of global health institutions is the Consortium of Universities for Global Health (CUGH). Even though only 16% of the world population lives in High-Income Countries (HICs) [21], 80.5% of the CUGH’s 195-member institutions are located in HICs, while the remaining 19.5% are in LMICs [22]. In other words, global health curricula and pedagogy has been mostly limited to HICs. In response to this divergence, in 2010, the International Federation of Medical Students, representing 1.2 million medical students from 123 countries, urged medical schools globally to offer more global health courses within their curriculum [23].

In response to this demand, and growing health challenges in Mexico, and globally, in 2019, the Medical and Health Sciences School at Tecnológico de Monterrey (Tec) launched the first multidisciplinary online global health course in the country: “Global Health for Leaders.” Tec is a high-ranked, private university in Mexico with approximately 90,000 students, 31 campuses, and 10,000 professors [24], and its Medical and Health Sciences School is a nationally acclaimed pioneer in the field of global health. Since 2010, Tec’s Medical and Social Sciences School has taught and organized global health courses and conferences. Notably, the global health course was created as part of the new educational approach called Model Tec21 which aims to hone the abilities of its graduates and develop competencies required to tackle the challenges of the twenty-first century [25].

Uniquely, this course sought to enroll students from all of Tec’s campuses and disciplines and used a competency-based approach, recommended by the CUGH. The first course consisted of 153 students, 30 were from the health-sciences and 123 from other disciplines, including architecture, finance, business and management, engineering, and marketing. The course was the best-ranked online course at Tec in the fall of 2019. Following the good ratings, in the Spring of 2020, 348 students from different disciplines and schools enrolled. The course consisted of regular zoom sessions, team-based activities, reflective writing assignments, and a final individual project that prompted making a TED-style video addressing a global health challenge in Mexico (For details see the syllabus in the Additional file 1: Appendix). The course challenged students to delve into the complexities of global health at both a theoretical and practical level while tackling some of the most imminent health challenges we face today. As the course was unprecedented in Mexico, and we have not read of a similar effort in a LMIC, we decided to evaluate the course (Refer to Additional file 1: Appendix for a list of the domains and competencies aimed to be developed during the course).

Our study responds to the question: Is it possible to develop global health recommended competencies in an online course offered to undergraduates? To respond to this question we used student responses to a pre and post-course survey aimed to evaluate self-perceived competencies in global health.

## Methods

### Setting and sample population

We conducted a cross-sectional study with a pre- and post-design. Students at the Tec enrolled in Global Health for Leaders in the fall of 2019 (*n* = 153) and the spring of 2020 (*n* = 348) were selected for this study. Our study population included students from 17 campuses located in 16 states and in Mexico City, and academic disciplines such as medicine, engineering, business, architecture, and psychology (See Table 1 with the characteristics of the participants). We had an overall 91% survey response in the pre-course survey and 87% in the post-course survey.

**Table 1:** Demographic characteristics of the study participants

Demographic characteristics of the participants enrolled in the course	No. (%)
**Gender**	
Female	234 (51.3)
Male	222 (48.7)
**Campus**	
Monterrey	167 (36.6)
Mexico City	62 (13.6)
Santa Fe	56 (12.3)
Guadalajara	43 (9.4)
State of Mexico	24 (5.2)
Toluca	21 (4.6)
Tampico	19 (4.1)
Saltillo	17 (3.7)
Laguna	10 (2.6)
Queretaro	9 (2)
Aguascalientes	7 (1.5)
Puebla	6 (1.3)
Leon	5 (1.1)
Irapuato	4 (0.9)
Morelia	3 (0.65)
San Luis Potosi	2 (0.4)
Zacatecas	1 (0.2)
**Discipline of study**	
Business	210 (46)
Engineering and Science	138 (30.3)
Health Sciences	60 (13.2)
Humanities	30 (6.6)
Social Sciences	18 (3.9)

### Survey development

The content of the survey instrument was informed by the Interprofessional Global Health Competencies framework recommended by the CUGH [26]. We selected seven global health competencies recommended for a global health citizen level which includes students and professors from any discipline. The questionnaire covered competencies in the domains of Global Burden of Disease, Globalization of health and health care, Social and Environmental Determinants of Health, Collaboration, Partnering, Communication, Ethics, Professional Practice, and Health Equity and Social Justice (See Additional file 1: Appendix for the competencies assessed in each domain). The estimated completion time was 2–4 min. Students were asked to rate various competencies endpoints on an ordinal-five-point scale (strongly agree to strongly disagree). Other information such as the discipline of study and level of knowledge about global health before the course on an ordinal-four-point scale (“I don’t know anything” to “I know the subject well”) was also obtained. In addition, we included two open-ended questions in the post-course survey: “What was the most important thing that you learned in this course?” And “What advice do you have to improve the course?”

### Data collection

The survey was self-administered before starting the course in the fall in August 2019 and ending the course in December 2019, then during the spring course in February 2020 and June 2020. This pre-post design was intended to assess the effects of the course on the self-perceived competencies acquired by the students. We chose an identical point in the academic cycle in both years to avoid the potential confounding effects of academic cycle variation in students’ burden [27]. We circulated the survey by e-mail and students’ WhatsApp groups, and informed the students that their responses were confidential and would not have any effect on their grades. We did not offer any incentives to encourage responses.

### Data analysis

All data from both surveys were collected using Redcap®. We performed descriptive statistical analyses using STATA® Version 14, comparing the combined pre-tests (from Fall and Spring cohorts) to the combined post-tests. Fisher’s exact test was used to compare the samples with statistical significance reached at *p*-value< 0.05. We also disaggregated and analyzed the samples by gender and students’ field of study. In addition, sensitivity analyses were performed by comparing students who took the course in fall 2019 versus students who took the course in spring 2020, without substantial differences, and our sample was of sufficient size to detect meaningful differences with this methodology.

## Results

Of the 501 pre-course surveys administered, a total of 456 responses were completed - 139 from fall 2019 and 317 from spring 2020. Of the 501 post-course surveys administered, a total of 435 were completed − 133 from fall 2019 and 302 from spring 2020. The combined pre-course survey response rate was 91%, and the post-course response rate was 87% with an overall response rate of 88.9%. The demographics for the 2019 and 2020 course cohorts are displayed in Table 1. Of the students enrolled, 51.3% (234/456) were female. More than half of the students in both cohorts attended the Monterrey (36.6%) and Mexico City (13.6%) campus locations. This course successfully enrolled students from a myriad of professional tracks, and not exclusively students in the health sciences. The majority (46%, 210/456) of the students enrolled in the course studied business or in a business-related field (e.g. economics, finance, or international affairs) and engineering sciences (30.3%, 138/456). Only 13.2% (60/456) of the students enrolled were in the medical sciences (Table 1).

In addition, most of the participants (73%) responded in the pre-course survey that they knew little about the topics in global health and the field in general. Conversely, in the post-course survey, 85% of the students felt that they knew the topic well or dominated its varied elements (*p* < 0.001) (Table 2). Further, only 8.7% of the respondents in the pre-course survey strongly agreed that they could describe fundamental aspects of global health such as the Millennium Development Goals or Sustainable Development Goals, in contrast to a 56% of the students who strongly agreed in the post-course survey (*p* < 0.001) (Table 2). In addition, only 13% of students in the pre-course survey strongly agreed that they could describe the negative and positive effects of globalization on health, in contrast to 50% in the post-course survey (*p* < 0.001). More than three-quarters of students in the post-course cohort either strongly agreed or agreed that they could describe how healthcare systems function in varying countries, compared to less than one-quarter in the pre-course cohort (*p* < 0.001). Moreover, only 17.9% of the students strongly agreed in the pre-course survey that they could describe how non-medical or non-biological factors such as socioeconomic and political forces affect health. After the course, this number increased to 49% (*p* < 0.001) (Table 2).

**Table 2:** Results of self-perceived global health competencies in the pre and post-course survey

Question	Levels	Pre-course ***N*** = 456	Post-course ***N*** = 435	Differences between pre-and post-course percentages (***p***-value from Fisher’s exact test)
How much do I know about global health	I dominate the topic well	0.6%	20%	< 0.001
	I know the topic well	8.5%	65%	
	I know the topic a little bit	73%	13%	
	I don’t know what it is	17%	1.3%	
“I feel comfortable explaining the main causes of death and disease in the world”	Strongly Agree	27%	45%	< 0.001
	Agree	37%	49%	
	Neutral	21%	3.4%	
	Disagree	11%	0.6%	
	Strongly disagree	3.7%	2%	
“I can describe health challenges at a global and national scale”	Strongly Agree	12%	55%	< 0.001
	Agree	40%	39%	
	Neutral	30%	4%	
	Disagree	14%	0.7%	
	Strongly disagree	4%	2%	
“I can describe how non-biological and non-medical factors (political, social, economic, and environmental) are related to health and disease”	Strongly Agree	17.9%	49%	< 0.001
	Agree	48%	43%	
	Neutral	21%	5%	
	Disagree	10%	0.2%	
	Strongly disagree	2.4%	2.3%	
“I can describe the fundamental aspects of global health efforts (e.g. Millennium Development Goals, Sustainable Development Goals)	Strongly Agree	8.7%	56%	< 0.001
	Agree	26%	37%	
	Neutral	34%	3.9%	
	Disagree	23%	0.9%	
	Strongly disagree	7%	2.3%	
“I can describe the positive and negative effects of globalization on health”	Strongly Agree	13%	50%	< 0.001
	Agree	46%	40%	
	Neutral	24%	6%	
	Disagree	14%	1.2%	
	Strongly disagree	3%	2%	
“I feel comfortable describing the principal objectives and functions of a healthcare system”	Strongly Agree	17%	41%	< 0.001
	Agree	35%	45%	
	Neutral	24%	10%	
	Disagree	18%	2%	
	Strongly disagree	6%	1%	
“I can describe how healthcare systems function in other countries”	Strongly Agree	7.7%	33%	< 0.001
	Agree	16%	44%	
	Neutral	31%	16%	
	Disagree	31%	5%	
	Strongly disagree	14%	1.7%	
“I understand the relationship between human rights, inequities, and health”	Strongly Agree	18%	49%	< 0.001
	Agree	39%	43%	
	Neutral	27%	5%	
	Disagree	1%	2%	
	Strongly disagree	4%	1%	
“I can apply topics in justice, equity, and human rights to global health problems”	Strongly Agree	19%	53%	< 0.001
	Agree	39%	41%	
	Neutral	25%	7%	
	Disagree	11%	0.2%	
	Strongly disagree	5%	1.5%	

The interdisciplinary nature of the course encourages students to understand the interconnectedness between health and a myriad of fields. While only 18.9% of students in the pre-course strongly agreed to have an understanding of the relationship between human rights, inequities, and health, almost half (49%) of the students in the post-course cohort strongly agreed with the statement (*p* < 0.001). Similarly, only 19% of pre-course students strongly agreed that they could apply the aforementioned topics to global health problems while half of the students (53%) responded strongly agreed to the same statement in the post-course survey (*p* < 0.001). When we analyzed the samples by gender, we did not notice any difference in the effect of the class. However, when we analyzed the pre and post-test samples by students’ field of study, we did notice some variation in regards to previous understanding of global health matters between medicine students and other fields (*p* = 0.03); however, the effect of the course in terms of percentage change was very similar and statistically significant across fields of study.

In the survey we also included questions on 1) course expectations, 2) main takeaways, and 3) recommendations to improve the course.

**Fig. 1:**
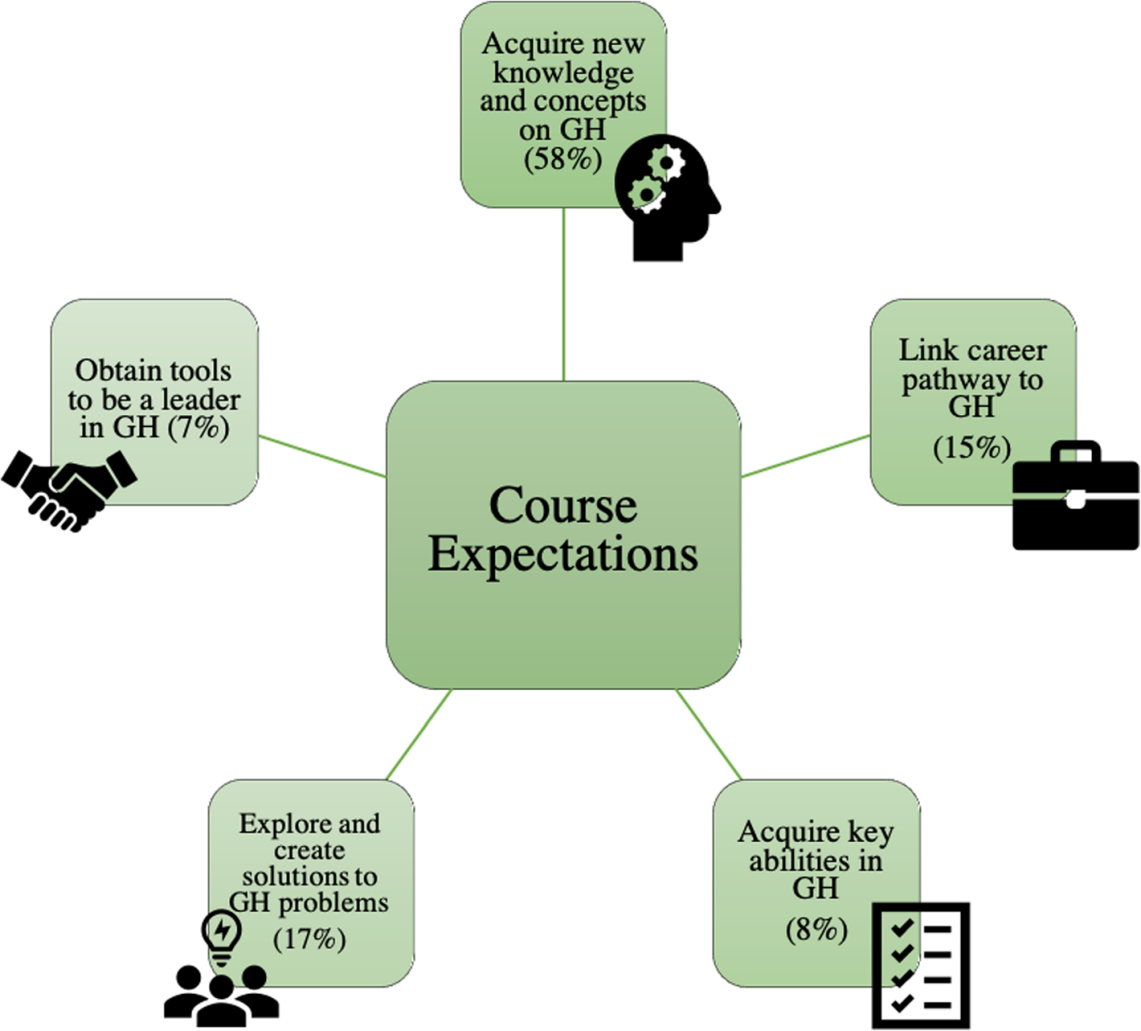
Student course expectations in the pre-course survey for both 2019 and 2020 cohorts (*N* = 163)

Students were asked to include individual comments about their expectations prior to commencing the course. Out of the students who received the survey, 163 students completed the open response section (Fig. 1). Students most commonly expected to obtain new knowledge pertaining to the field of global health (58%). Other responses included, explore solutions to GH problems (17%), link current career to the field of GH (15%), gain the tools necessary to become a leader in the field of GH (7%) and acquire key abilities to work and solve problems in the field of GH (3%).

**Fig. 2:**
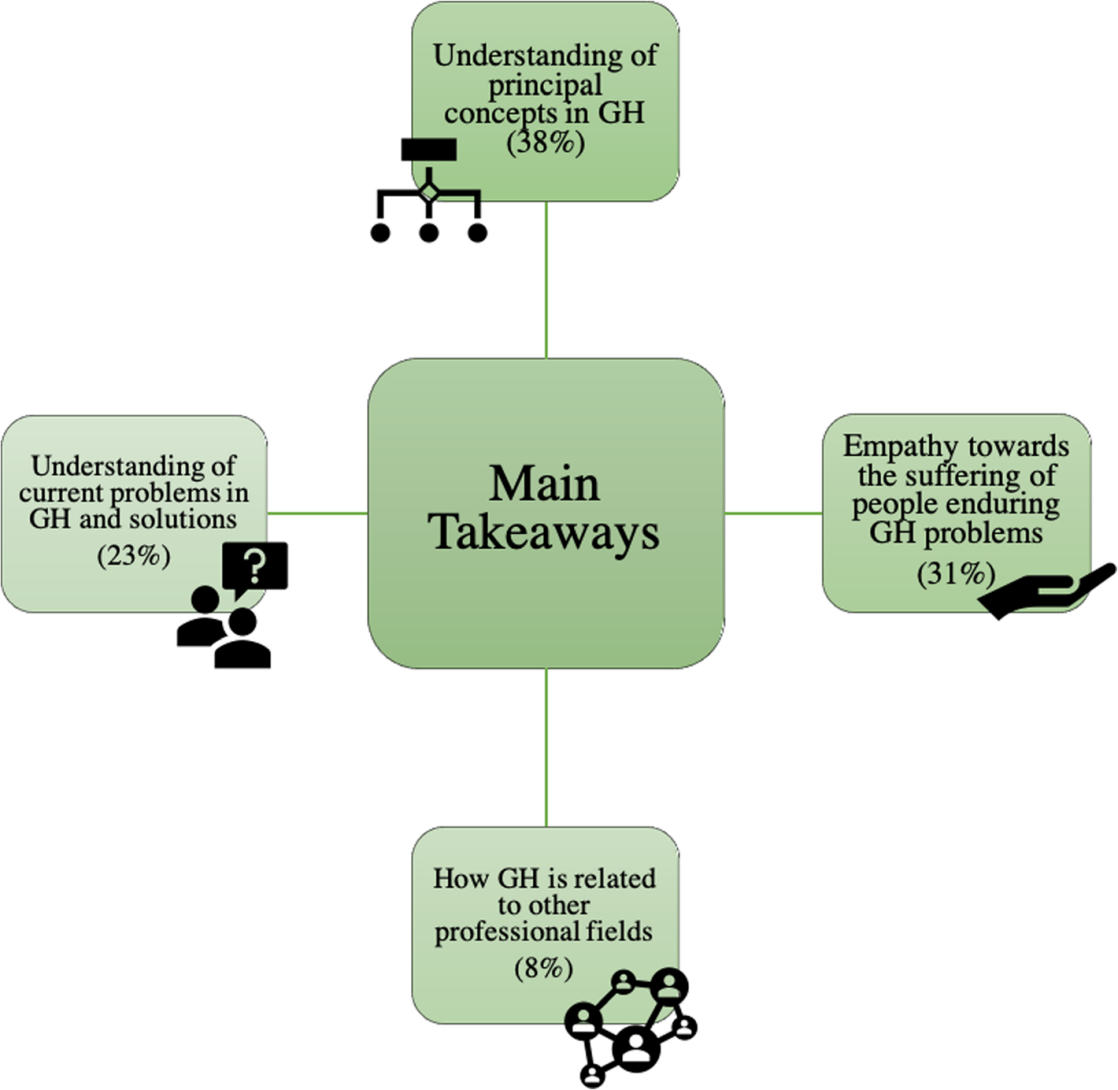
Student course takeaways in the post-course survey for both 2019 and 2020 cohorts (*N* = 104)

The post-course survey asked students to add their main takeaways in the course, and a total of 104 responses were received (Fig. 2). The majority of the students (38%) responded that they acquired understandings of principal concepts in global health such as common diseases, social determinants of health, common metrics of disease, and health systems. However, this was not one of their expectations, students also felt that the course helped them develop a more empathetic perception of the suffering of others experiencing global health-related issues (31%). 23% also mentioned that they gained a better understanding of current global health issues and their solutions (23%). Finally, other students felt that the course helped them comprehend the interdisciplinary nature of global health (8%). Below are some quotes from the survey responses divided by common areas.

Students noted that health includes broader topics beyond biological and clinical sciences, and their associated competencies encourage them to take active roles:

*“I learned that health should always be considered regardless of career pathways since there are global problems where everyone has the opportunity and the responsibility to help.” (Student from the Monterrey campus).*

Moreover, students indicated that the course helped them have empathy towards others’ suffering and also faced narrow explanations for disease, such as the approach of blaming the victim by using a behaviorist approach that assumes that decisions in health are not linked to contextual factors but are a solely individual responsibility [28].

*“I learned about the importance of empathy while working with vulnerable populations since disease and poverty is not entirely an individual’s fault; there are many factors and structures that are determinants of social conditions.”(Student from the Mexico City campus).*

Also, the course helped students gain knowledge in core areas such as the global burden of disease and social determinants of health, and helped them reflect on potential solutions.

*“I learned to identify different causes of mortality and suffering in the world in addition to frameworks helpful in proposing novel ideas to improve global health.” (Student from the Guadalajara campus).*

*“This course opened my eyes to the pervasive health problems that impact communities on a global scale, as well as a myriad of solutions to combat and prevent these problems.” (Student from the Toluca campus).*

Finally, the course helped students think about healthcare and health systems as an organized response to mitigate and prevent disease and human suffering, which is very important to their role as agents of health and responsible citizens keeping health institutions accountable.

*“I learned about the importance of health systems in a country given that it is a central social support system for citizens, hence it is how governments can improve wellbeing and development.” (Student from the Chihuahua campus).*

**Fig. 3:**
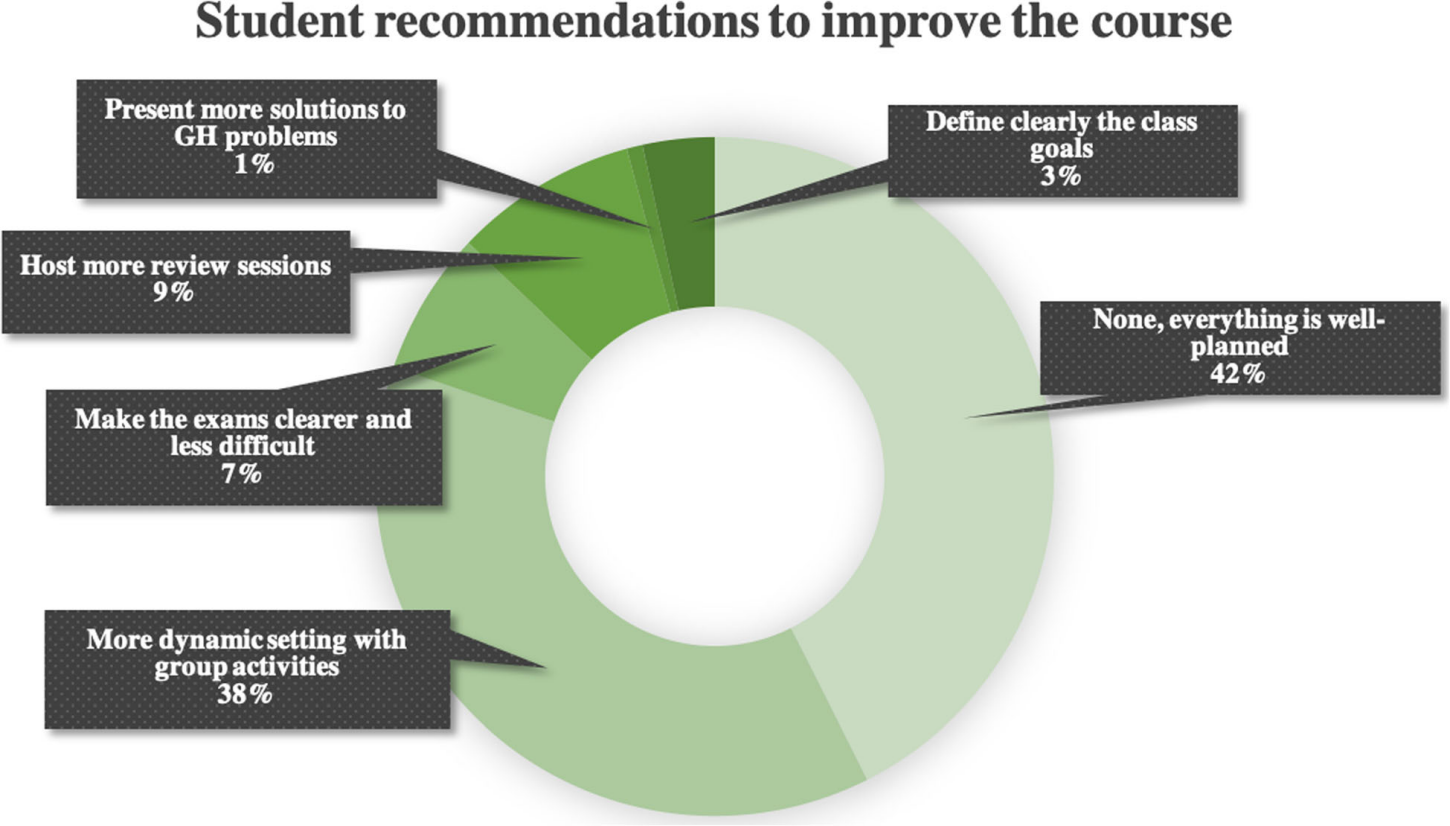
Student recommendations to improve the course

Students also advised on areas for improvement in the post-course surveys (See Fig. 3). The majority of students (42%) indicated that there was little to no changes needed in the course, and it was generally well-planned and conducted. 38% of the students suggested a more dynamic teaching environment and would have liked to see more interactive material and engaging activities throughout the course. Other responses included having more review sessions (9%), clearer exam formats (7%), well-defined objectives at the beginning of each week (3%), and developing more solutions to current GH problems (1%).

## Discussion

Global health problems manifest locally, and require contextualized solutions, hence authors and global health experts have been advocating to expand Global Health Education (GHE) to LMICs, but still, there are not many examples. Further, there is no substantial evidence on how to develop global health competencies in an LMIC context. The value of our results relies on exemplifying that institutions in an LMIC can teach GHE courses focused on developing global health competencies to address global health issues locally. As a whole, the students enrolled in our course perceived a significant change in their understanding of critical global health topics, including social determinants of health, health systems, globalization, burden of disease, multilateral organizations, and global health solutions. This point is further solidified when considering that nearly all students (85%) indicated that they dominated or knew well these topics in the post-course survey, compared with 9. 1% in the pre-course survey (Table 2).

Our course successfully met students’ initial expectations, for example, 58% of students expected to acquire new knowledge pertaining to the field of global health and, in the post-course survey 38% indicated that their main takeaway was a comprehensive understanding of GH topics (Figs. 1 and 3, respectively). Similarly, 17% of students expected to explore and create solutions to GH problems, and 23% felt that they gained an understanding of solutions to global health dilemmas (Figs. 1 and 3, respectively). Incorporating student feedback is crucial to the learning process, particularly in an online learning format. Studies have shown that feedback can strengthen student learning and understanding, and promote using course material beyond the scope of the course [29]. At the beginning of a global health course, we propose conducting a pre-course survey to identify student goals and expectations, and adapt the curriculum to accomplish these. After the course, analyze if student goals were met using a post-course survey.

A cornerstone of global health is the development of empathy towards suffering, also called pragmatic solidarity, a philosophical standpoint that aims to walk in solidarity with those who suffer while trying to find practical solutions that account for the social, economic, and human rights of a person [30]. Figure 2 shows that 31% of the students shared that the main takeaway of our course on developing more empathy towards vulnerable populations in light of imminent and current global health problems. This empathy is needed everywhere, especially in LMICs, where disparities and inequities in health are rampant. Still, empathy is not enough, as some authors have discussed, and global health actors should link empathy to pragmatic solutions [18, 31, 32]. Moreover, GHE encourages students to actively focus on the relief of suffering in ways that are consonant with the vision of the vulnerable themselves with empathy for those in underserved conditions. How to translate that empathy towards pragmatic solidarity is a question we aim to answer with GHE.

Global health has been mostly relegated to medical students. In the United States, around 68% of medical schools have active global or international health interest groups [33] and the Association of American Medical Colleges data shows nearly half of all graduating medical students in 2005 participated in global health electives [34]. About 24% of U.S. medical schools offer a global health track, certificate, or concentration [34]. Increasing interest in our course at the Tec and our study shows that GHE is needed in undergraduate programs to educate more aware and empathic citizens. Moreover, undergraduates at Tec were compelled with the notion of global health and found the material useful for their careers. There is value in expanding GHE to other disciplines beyond the health sciences.

A critical characteristic of global health is its inter and transdisciplinary focus [20, 35]. Global health issues are complex and intricate thus requiring more than a single-discipline-approach. Hence, GH gathers information from multiple perspectives and disciplines to address pervasive global health issues. As shown in Table 2, our course helped students understand the interdisciplinary nature of health and global health. This is illustrated by a 31.1% increase of students who could describe the relationship between health and economic, political, social, and cultural factors. Moreover, students felt that they could apply topics from their respective fields to that of global health, as reflected in the open responses.

In a globalized and interdependent world, it is important that students are academically, professionally, and personally prepared to tackle imminent challenges as global citizens. Global citizenship academic frameworks are increasingly the norm in higher education [36], and a vital component to this global health course. By the end of the course, over 90% (Table 2) of students strongly agreed or agreed that they understood the effects of globalization on health, compared to an initial 59% (Table 2). As global citizens, students should be able to identify the main causes of disease on a global scale, and 94% of students in the post-course survey felt that they could describe major causes of death and disease, in contrast to a 64% in the pre-course survey.

The open-ended comments section of the survey revealed several limitations of the course and avenues for improvement. The course was offered solely online which hindered the dynamic nature of the activities. Some students felt that future iterations of the course should be more engaging. We believe that incorporating more group and practice-based activities in the syllabus would target this main concern. A majority of the students felt that the material was well planned; however, some students believed that routine review sessions would facilitate learning the material. This could also be targeted by clearly defining and stating the course objectives at the beginning of each course.

Although this study is unique in its focus, it has several limitations. The attrition rate from the pre-course to the post-course survey was 4%, which is low but could indicate self-selection bias. Since we were interested in the overall impact of the course, we used group estimates, instead of individual student responses from the pre and post-course surveys to evaluate individual student progress. Overall, the survey analyzed students’ perceived competencies; however, we did not assess these competencies in terms of attitudes, knowledge, or values [37]. Although we did observe an improvement in perceived global health knowledge, it is unclear how much of this knowledge can be effectively applied to global health problems or if perceived levels of empathy and solidarity were effectively improved. Future studies need to evaluate the knowledge-practice gap between the pre and post-course student feedback, especially in the context of an online global health course.

Online education will undoubtedly continue to grow at a rapid pace. In the backdrop of a pandemic that has affected all aspects of life, including education, online learning has stepped onto the forefront. There is ample evidence that online training can transmit knowledge and skills, especially when paired with clear goals and instruction [38]. However, there is no substantial evidence of the value of an online course in developing global health competencies. In our study, we discovered that 86% of students in the post-course survey felt prepared to describe key aspects of healthcare systems, in contrast to 52% of students (Table 2). Under the main takeaways, 32% of students felt that they understood how to properly address populations with empathy (Fig. 2).

Hence, online education might be an innovative force in the global health education movement. Based on our experience and feedback from our students, we have several recommendations to structure a successful global online course: 1) design the course to be accessible to students from diverse backgrounds, 2) incorporate dynamic activities where students interact with each other, 3) maintain at least one professor or teaching assistant per 20 students, 4) utilize an interactive platform to communicate with students and share timely relevant resources, we have used Remind® with satisfactory results, and 5) assign a final project as a culmination effort where students practice the competencies acquired.

## Conclusions

In an increasingly interconnected world, we need transformative pedagogy that enables students to resolve complex challenges concerning humanity. More than ever, LMICs need versatile, trans-disciplinary, and informed leaders working to find solutions to global health challenges and minimizing health risks. In this paper, we have presented our experience teaching an online global health course for multidisciplinary undergraduates in an LMIC. The competencies reported by our students indicate that the course prepared them to confront complex global health issues by helping them develop more empathy for the underserved, a greater understanding of contextual factors that influence health, greater ability to work in teams in inter- and transprofessional settings, and a stronger commitment to alleviate health disparities. Universities in LMICs have a unique opportunity to train these much-needed professionals to ensure that their communities benefit from a local cadre of global citizens committed to advance social justice, and health professionals trained to target the structural causes of disease.

## Supplementary Information

**Additional file 1.**

## Data Availability

The datasets used and/or analysed during the current study are available from the corresponding author on reasonable request.
