# Supplementary material for: Evaluation of a multidisciplinary global health online course in Mexico
Source: Glob Health Res Policy. 2020 Nov 17;5:48. doi: 10.1186/s41256-020-00179-8 (PMC7670103; doi:10.1186/s41256-020-00179-8)
Supplement: Supplementary file 1 — Additional file 1. [file 41256_2020_179_MOESM1_ESM.docx]

**Teaching Competencies in Global Health: Evaluation of an Online Course in Mexico**

**Appendix:**

**Global health Domains and competencies for a global citizen level. Competencies included are *italicized***

Domain 1: Global Burden of Disease:

· ***Competency 1a: Describe the major causes of morbidity and mortality around the world, and how the risk of disease varies with regions.***

***· Competency 1b: Describe major public health efforts to reduce disparities in global health (such as the Millennium Development Goals and the Global Fund to Fight AIDS, TB, and Malaria).***

· Competency 1c: Validate the health status of populations using available data (e.g., public health surveillance data, vital statistics, registries, surveys, electronic health records and health plan claims data).

Domain 2: Globalization of Health and Health Care

· ***Competency 2a: Describe different national models or health systems for provision of healthcare and their respective effects on health and healthcare expenditure.***

*· C****ompetency 2b: Describe how global trends in healthcare practice, commerce and culture, multinational agreements and multinational organizations contribute to the quality and availability of health and healthcare locally and internationally.***

***· Competency 2c: Describe how travel and trade contribution to the spread of communicable and chronic diseases***

· Competency 2d: Describe general trends and influences in the global availability and movement of health care workers.

Domain 3: Social and Environmental Determinants of Health:

· ***Competency 3a: Describe how cultural context influences perceptions of health and disease.***

***· Competency 3b: List major social and economic determinants of health and their impacts on the access to and quality of health services and on differences in morbidity and mortality between and within countries.***

*·* ***Competency 3c: Describe the relationship between access to and quality of water, sanitation, food and air on individual and population health.***

Domain 5: Collaboration, Partnering and Communication:

· Competency 5a: Include representatives of diverse constituencies in community partnerships and foster interactive learning with these partners.

· Competency 5b: Demonstrate diplomacy and build trust with community partners.

· Competency 5c: Communicate joint lessons learned to community partners and global constituencies.

· ***Competency 5d: Exhibit interprofessional values and communication skills that demonstrate respect for, and awareness of, the unique cultures, values, roles/responsibilities and expertise represented by other professionals and groups that work in global health.***

***· Competency 5e: Acknowledge one’s limitations in skills, knowledge, and abilities.***

· Competency 5f: Apply leadership practices that support collaborative practice and team effectiveness.

Domain 6: Ethics

· ***Competency 6a: Demonstrate an understanding of and an ability to resolve common ethical issues and challenges that arise in working within diverse economic, political and cultural contexts as well as working with vulnerable populations in low resource settings to address global health issues.***

***· Competency 6b: Demonstrate an awareness of local and national codes of ethics relevant to one’s working environment.***

· Competency 6c: Apply the fundamental principles of international standards for the protection of human subjects in diverse cultural settings.

Domain 7: Professional Practice:

· ***Competency 7a: Demonstrate integrity, regard and respect for others in all aspects of professional practice.***

***· Competency 7b: Articulate barriers to health and healthcare in low-resource settings locally and internationally.***

· Competency 7c: Demonstrate the ability to adapt clinical or discipline-specific skills and practice in a resource-constrained setting.

Domain 8: Health Equity and Social Justice

· ***Competency 8a: Apply social justice and human rights principles in addressing global health problems.***

· Competency 8b: Implement strategies to engage marginalized and vulnerable populations in making decisions that affect their health and well-being.

· ***Competency 8c: Demonstrate a basic understanding of the relationship between health, human rights, and global inequities.***

· Competency 8d: Describe the role of WHO in linking health and human rights, the Universal Declaration of Human Rights, International Ethical Guidelines for Biomedical Research involving Human Subjects.

· ***Competency 8e: Demonstrate a commitment to social responsibility.***

· Competency 8f: Develop understanding and awareness of the health care workforce crisis in the developing world, the factors that contribute to this, and strategies to address this problem.

Domain 10: Sociocultural and Political Awareness

· Competency 10a: Describe the roles and relationships of the major entities influencing global health and development.

· ***Competency 11a: Identify how demographic and other major factors can influence patterns of morbidity, mortality, and disability in a defined population.***

Syllabus: Global Health for Leaders

**Associated discipline:**

Health Sciences

**School:**

Medicine and Health Sciences

**Academic Department:**

Medical Sciences

**Competencies:**

- Responsibility as a global citizen
- Critical understanding of complex issues
- Communication

**Prerequisites:**

None

**Course objectives:**

This is a basic level course intended for students to develop a conceptual and strategic framework on global health, recognizing its link to sustainable human and social development. The student will be able to make an evaluative and purposeful report identifying areas of opportunity and challenges that currently exist in the field of global health.

**General Objectives of the Training Unit:**

At the end of the training unit the student will:

- Define the basic concepts of global health and identify aspects that distinguish it from other forms of health care

- Recognize determinants of public health and their relationship with indicators of human development and wealth, in a globalized context

- Analyze the specific weight of the most common pathologies and their incidence in local, national and global health systems.

- Identify current and foreseeable challenges in global health

**Course Content:**

**Section I:** Health in the global environment.

1. Historical framework.

1.1 Nature, man and society: historical paradigms.

1.2 Evolution of the concepts of health and disease.

1.3 Development of Health Sciences. Scientific medicine versus non-validated medicine.

1.4 The right to health in the global environment.

2. Fundamentals of global health.

2.1 Definitions and basic concepts.

2.2 Importance of health in a global context.

2.3 Social determinants of health.

2.4 Main health problems of today.

3. Sustainable Development Goals (ODs).

3.1 Background to the MDGs and the new Millennium Goals.

3.2 Sustainable Development Goals by 2030.

3.3 Social responsibility for sustainability.

4. Epidemiological transition and health indicators.

4.1 Demographic and epidemiological transition.

4.2 Public health indicators.

4.3 Demographic and epidemiological trends in the coming decades.

4.4 Primary health care.

5. Health systems in the global environment.

5.1 Main functions of a health system.

5.2 Organization and financing of a health system.

5.3 Effectiveness and efficiency of a health system.

5.4 Health systems in high, medium and low-income countries.

**Section II:** Biomedical and Socio-Environmental Aspects of Global Health

6. Maternal and child health.

6.1 Main causes of maternal and child mortality in Mexico and the world.

6.2 Vulnerable populations.

6.3 Preventive measures undertaken at the global level.

6.4 Priority strategies in the coming decades.

7. Communicable diseases.

7.1 Communicable diseases with the greatest global impact in the first decades of the 21st century.

7.2 Vulnerable populations.

7.3 Preventive measures undertaken at the global level.

7.4 Priority strategies in the coming decades.

8. Non-communicable diseases.

8.1 Non-communicable diseases with the greatest global impact in the first decades of the 21st century.

8.2 Vulnerable populations.

8.3 Preventive measures undertaken at the global level.

8.4 Priority strategies in the coming decades.

9. Mental and elderly health.

9.1 Impact of mental health in the workplace and society

9.2 Identify vulnerable groups and preventive measures

9.3 Define the main problems of the elderly populations

9.4 Measures for the inclusion of the elderly in the labor and social sectors

9.5 Establish advantages and disadvantages between different health systems.

10. Environmental health and safety.

10.1 Global warming and its consequences.

10.2 Primary objectives of the Paris Agreement.

10.3 Vulnerable populations by continent.

10.4 Preventive measures undertaken at the global level

10.5 Priority strategies in the coming decades.

11. Food security and clean water.

11.1 Impact of adequate nutrition on different age groups.

11.2 Indicators of adequate nutrition.

11.3 Vulnerable populations.

11.4 Priority strategies for the coming decades.

12. Migration.

12.1 Global impact.

12.2 Impact on Mexico.

**Section III:** Transforming global health.

13. Challenges in global health in the new millennium.

13.1 Science and technology.

13.2 Ethical implications and human rights in global health.

13.3 International cooperation and partnerships to optimize health.

13.4 Leadership and empowerment of global health actors

13.5 Social entrepreneurship strategies.

**Specific learning objectives by subject:**

Teaching methodology and learning activities:

LEARNING ACTIVITIES UNDER THE LEADERSHIP OF AN ACADEMIC

1. Review the theoretical framework that support the key themes of the course on the concept of global health and the context of preventive medical care in a globalized framework, with a primary emphasis on sustainable human and social development.
2. Analysis and discussion of cases, while establishing close understanding of the key topics of the course.
3. Development and presentation of proposals on a selected topic.

INDEPENDENT LEARNING ACTIVITIES

1. Self-directed study and critical reading of the topics and subtopics of the course on the concept of global health in the context of preventive medical care in a globalized framework, with a primary emphasis on sustainable human and social development.
2. Preparation of topics and cases to present in course related to the key topics of the course.
3. Individual and collaborative activities that aim to develop key competencies related to the course objectives

**Suggested teaching technique:** Collaborative learning

**Estimated time of each topic:**

Topic 1 4 hours

Topic 2 4 hours

Topic 3 4 hours

Topic 4 4 hours

Topic 5 4 hours

Topic 6 4 hours

Topic 7 4 hours

Topic 8 4 hours

Topic 9 4 hours

Topic 10 4 hours

Topic 11 4 hours

Topic 12 4 hours

Topic 13 8 hours

Evaluation 4 hours

**Total 60 hours**

**Suggested evaluation criteria:**

To evaluate student learning, the following criteria will allow the teaching staff to gauge the students learning progress in the course. The grading rubric is as follows:

- **20% --- Activities.** Active participation in debates, discussions and quick exams will be evaluated with a particular focus on student comprehension and application of key concepts discussed in the course.
- **50% --- Online exams.** Exams will test the student’s comprehension and analytical capabilities relating to the course objectives on global health focusing on preventive medical care and globalized frameworks, and with an emphasis on sustainable human and social development (2 exams).
- **30% --- Final project.** A proposal on a global health challenge is evaluated with an accompanying presentation.

**Suggested Bibliography:**

TEXTBOOKS:

- Skolnik, Richard L., Global health 101, Burlington, MA: Jones and Bartlett Learning, 2019, eng, 1449675530, 9781449675530

REFERENCE BOOKS:

- Babulal Sethia, Parveen Kumar, Essentials of Global Health, Elsevier Health Sciences, 2018, 0702066087, 9780702066085
- Debra L. DeLaet, David E. DeLaet, Global Health in the 21st Century: The Globalization of Disease and Wellness, Routledge, 2015, 1317258991, 9781317258995

Teacher Profile:

- Masters in International Studies; Masters in Social Sciences; Masters in Health Sciences; Masters in Public Health; Surgeon Doctor with Specialty in some field of Medicine; Doctorate in International Studies; Doctorate in Social Sciences; Doctorate in Health Sciences; (512201) Doctorate in Public Health

**Course language:**

- Spanish
